# CETP and APOA2 polymorphisms are associated with weight loss and healthy eating behavior changes in response to digital lifestyle modifications

**DOI:** 10.1038/s41598-023-48823-w

**Published:** 2023-12-07

**Authors:** Meelim Kim, Seolha Lee, Eun Cho, Kyung-Won Hong, So-Jin You, Hyung Jin Choi

**Affiliations:** 1 Department of Biomedical Science, Seoul National University College of Medicine, 28 Yungun-Dong, Chongno-Gu, Seoul, 110-799 Korea; 2 Herbert Wertheim School of Public Health and Human Longevity Science, University of California, San Diego, La Jolla, CA USA; 3 Center for Wireless and Population Health Systems, Calit2’s Qualcomm Institute, University of California, San Diego, La Jolla, CA USA; 4 The Design Lab, University of California, San Diego, La Jolla, CA USA; 5 Department of Global Medical Science, Sungshin Women’s University, Seoul, Korea; 6 Theragen Bio, Inc., Seongnam, Korea

**Keywords:** Genotype, Therapeutics, Weight management

## Abstract

Response to digital healthcare lifestyle modifications is highly divergent. This study aimed to examine the association between single nucleotide polymorphism (SNP) genotypes and clinical efficacy of a digital healthcare lifestyle modification. We genotyped 97 obesity-related SNPs from 45 participants aged 18–39 years, who underwent lifestyle modification via digital cognitive behavioral therapy for obesity for 8 weeks. Anthropometric, eating behavior phenotypes, and psychological measures were analyzed before and after the intervention to identify their clinical efficacy. *CETP* (rs9939224) *SNP* significantly predict “super-responders” with greater body mass index (BMI) reduction (p = 0.028; GG − 2.91%, GT − 9.94%), while *APOA2* (rs5082) appeared to have some potential for predicting “poor-responders” with lower BMI reduction (p = 0.005; AA − 6.17%, AG + 2.05%, and GG + 5.11%). These SNPs was also associated with significant differences in eating behavior changes, healthy diet proportions, health diet diversity, emotional and restrained eating behavior changes. Furthermore, classification using gene–gene interactions between rs9939224 and rs5082 significantly predicted the best response, with a greater decrease in BMI (p = 0.038; − 11.45% for the best response group (*CEPT* GT/TT × *APOA2* AA) vs. + 2.62% for the worst response group (*CEPT* GG × *APOA2* AG/GG)). *CETP* and *APOA2* SNPs can be used as candidate markers to predict the efficacy of digital healthcare lifestyle modifications based on genotype-based precision medicine.

**Trial registration:** NCT03465306, ClinicalTrials.gov. Registered March, 2018.

## Introduction

Obesity is a complicated disorder as its occurrence depends on various factors, such as dietary intake, energy expenditure, psychological features, and heritable factors^1,2^. Recently, genome-wide association studies (GWAS) have identified a large number of single nucleotide polymorphisms (SNPs) associated with obesity phenotypes^3^. One of the commonly used phenotypes to assess obesity is the body mass index (BMI) and it is known that approximately 40–70% of inter-individual differences in BMI are associated with genetic factors^4^. In addition, several previous studies have stated that there are a number of gene-diet interactions associated with changes in anthropometric and metabolic measures^5,6^. Because behavioral and psychological factors have been identified as important areas of research in obesity^7^, several studies also examined at the relationship between eating behavior and genes related to obesity. For example, it has been reported that pathological eating behaviors (e.g., Binge Eating Disorder, Anorexia Nervosa) are associated with both *NUCB2* rs757081 and *FTO* rs9939609^8^. Furthermore, a prior clinical trial found that CLOCK 3111T/C SNP interacts with emotional eating behavior to influence total weight loss^9^. Although the prevalence of obesity depends on multi-dimensional components, most of the current literature reviews the associations between obesity candidate genes and single-dimensional phenotypes such as anthropometrics or specific diet^10,11^.

With the increasing prevalence of obesity, various types of obesity interventions have also been developed. Nevertheless, obesity interventions are not successful for all individuals, suggesting that genetic traits contribute to the variability in weight loss in response to each type of intervention. This suggests that a personalized approach based on individual characteristics, such as genetics, is required to effectively treat obesity. In a previous study, the degree of weight loss after exercise was found to be more similar between identical twin pairs when compared to dizygotic twin pairs^12–14^. Moreover, the variation in genetic risk score (GRS) for lean body mass (LBM) may affect appetite changes and body composition in response to dietary fat intake^15^. Several clinical trials in the previous studies reported some specific *FTO*-diet intervention interactions^16,17^ which offers support to the idea that genetic variability should be taken into account when developing more successful weight-loss strategies. However, there have been no studies that investigated SNP genotypes that modulate the clinical outcomes in response to digital therapeutics for obesity.

**Figure 1:**
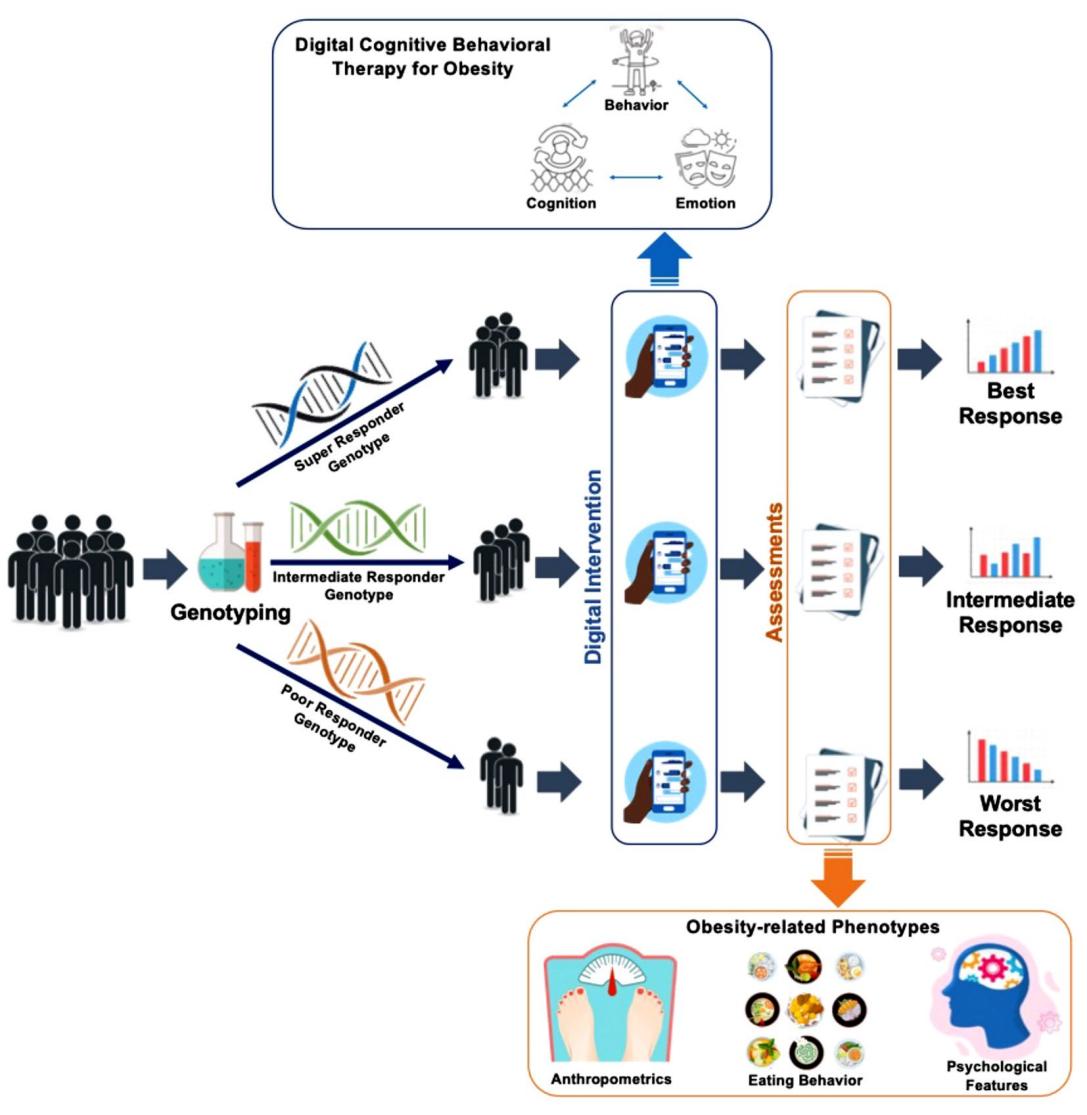
Diagram representing the use of precision medicine in Digital Cognitive Behavioral Therapy for Obesity (dCBT-O).

Herein, we aimed to examine the associations between SNP genotypes and clinical efficacy using multi-dimensional components of digital therapeutics (Fig. 1). We hypothesized that clinical outcomes, including anthropometrics, obesity-related behavioral phenotypes, and psychological characteristics, may differ in response to the digital lifestyle modification, called digital cognitive behavior therapy for obesity (dCBT-O), according to the candidate SNPs for obesity phenotypes. Thus, the candidate SNPs were investigated in order to identify which genotype modulated the changes in clinical outcomes in response to dCBT-O.

## Results

### Overall results from prior studies

The general characteristics and the results regarding anthropometrics of the participants have been reported in elsewhere^18^. In summary, the mean percentage weight loss of participants in the dCBT-O group after 8 and 24 weeks was 3.1% and 3.7% of their initial weight, respectively. These results support the conclusion that the dCBT-O group exhibited a legacy effect even after the intervention was terminated. In addition, healthy dietary intake and diversity were shown to be promoted after dCBT-O. We found that the major predictors of clinical efficacy were depression, anxiety, self-esteem, motivation, restrained eating behavior, body shape satisfaction, and insulin resistance. Here, we aimed to investigate the role of genetic polymorphisms in the prediction of weight loss and related phenotypes.

### Genotype frequency

In the present study, the genotype frequency stratified by each SNP, cholesterol ester transfer protein (CETP) rs9939224, and apolipoprotein A-II (APOA2) rs5082 genotypes are presented in Table 1. No significant deviation from HWE was observed (*p* > 0.05).

**Table 1:** Genotype and allele frequencies.

CETP rs9939224	G/G	G/T	T/T	*p*-value
N	37	8		
Genotype frequency	82.22%	17.78%		> 0.05

APOA2 rs5082	A/A	A/G	G/G	*p*-value
N	36	8	1	
Genotype frequency	80.00%	17.78%	2.22%	> 0.05

### Associations with CETP rs9939224 and phenotypes

**Figure 2:**
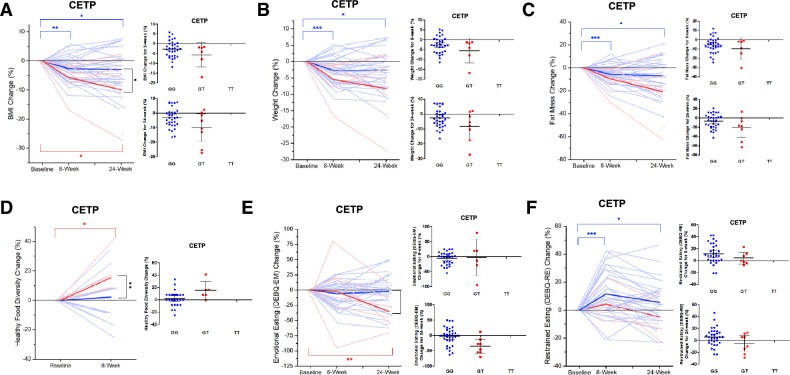
Association between *CETP* rs9939224 and obesity-related phenotypes.

Regarding the primary outcome, the *CETP* GG genotype was shown to lead to a − 2.62% (*p* = 0.001) and − 2.91% (*p* = 0.020) variance in BMI after 8 and 24 weeks, respectively. The *CETP* T allele was shown to lead to a − 5.68% (*p* = 0.067) and − 9.94% (*p* = 0.042) variance in BMI after 8 and 24 weeks, respectively. Moreover, the *CETP* T allele was significantly associated with a greater BMI decrease at 24 weeks (*p* = 0.028; GG group BMI − 2.91%, GT group BMI − 9.94%; Fig. 2A). The *CETP* GG group exhibited a − 2.69% (*p* < 0.001) and − 2.56% (*p* = 0.029) change in weight after 8 and 24 weeks, respectively, while the *CETP* T allele group exhibited a − 5.48% (*p* = 0.077) and − 8.24% (*p* = 0.053) change in weight, respectively. Thus, the changes in weight were shown to exhibit a decreasing trend at 24 weeks in the *CETP* T allele group (*p* = 0.052; GG group weight change − 2.56%, GT group weight change − 8.24%; Fig. 2B). Finally, we found that the *CETP* GG group exhibited a − 5.65% (*p* < 0.001) and − 7.26% (*p* = 0.010) change in fat mass after 8 and 24 weeks, respectively, whereas the *CETP* T allele group exhibited a − 9.70% (*p* = 0.091) and − 20.78% (*p* = 0.054) change in fat mass at 8 and 24 weeks, respectively. Accordingly, the *CETP* T allele group presented a decreasing trend in regards to fat mass at 24 weeks (*p* = 0.057; GG group fat mass change − 7.26%, GT group fat mass change − 20.78%; Fig. 2C).

When considering dietary behaviors, we found that the *CETP* T allele group exhibited a significantly improved healthy diet diversity after dCBT-O when compared to the *CETP* GG group (*p* = 0.007; GG group Healthy diet diversity change 2.15% (*p* = 0.372), GT group Healthy diet diversity change 15.27% (*p* = 0.048); Fig. 2D). In addition, the *CETP* GG group demonstrated a − 5.83% (*p* = 0.141) and − 2.65% (*p* = 0.603) variance in emotional eating behavior after 8 and 24 weeks, respectively, while the *CETP* T allele group showed a − 2.5% (*p* = 0.921) and − 35% (*p* = 0.008) variance in emotional eating behavior after 8 and 24 weeks, respectively. Accordingly, emotional eating behavior (DEBQ-EM) was shown to be significantly promoted in the *CETP* T allele group at 24 weeks when compared to the *CETP* GG group (*p* = 0.007; GG group DEBQ-EM − 5.83%, GT group DEBQ-EM − 35%; Fig. 2E). Lastly, the *CETP* GG group showed a + 11.47% (*p* < 0.001) and + 5.53% (*p* = 0.040) variation in restrained eating behavior (DEBQ-RE) after 8 and 24 weeks, respectively, whereas the *CETP* T allele group exhibited a + 4.12% (*p* = 0.346) and + 4.80% (*p* = 0.429) variation in restrained eating behavior after 8 and 24 weeks, respectively. The change in restrained eating behavior in the *CETP* T allele group exhibited an increasing trend when compared to the *CETP* GG group at 24 weeks (*p* = 0.091; GG group DEBQ-RE + 5.53%, GT group DEBQ-RE + 4.80%; Fig. 2F). Any of the results did not pass the strict multiple comparison corrected P threshold.

### Associations with APOA2 rs5082 and phenotypes

**Figure 3:**
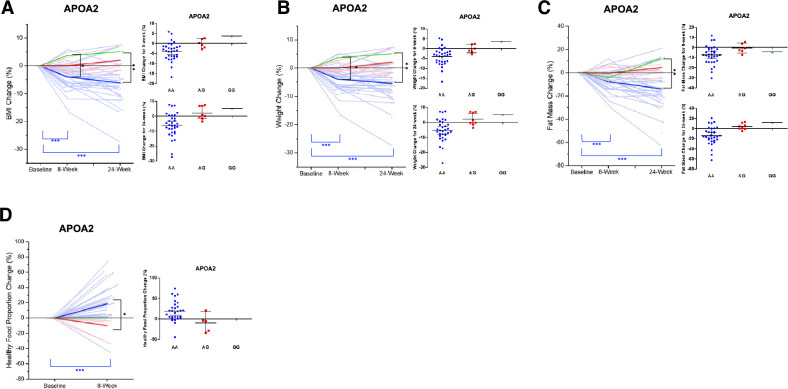
Association between *APOA2* rs5082 and obesity-related phenotypes.

According to our analysis, the *APOA2* AA group exhibited a − 3.93% (*p* < 0.001) and − 6.17% (*p* < 0.001) change in BMI after 8 and 24 weeks, respectively. The *APOA2* AG group showed a 0.02% (*p* = 0.980) and + 2.05% (*p* = 0.222) change in BMI after 8 and 24 weeks, respectively. The *APOA2* GG group showed a + 3.65% and + 5.11% change in BMI after 8 and 24 weeks, respectively. Thus, the *APOA2* G allele was shown to be significantly associated with an increasing trend in BMI (*p* = 0.012 and *p* = 0.005 at 8 and 24 weeks, respectively; Fig. 3A). In addition, the *APOA2* AA group showed a − 3.96% (*p* < 0.001) and − 5.39% (*p* < 0.001) change in weight at 8 and 24 weeks, respectively. On the other hand, the *APOA2* AG group showed a − 0.03% (*p* = 0.967) and − 2.14% (*p* = 0.200) change in weight at 8 and 24 weeks, respectively, while the *APOA2* GG group exhibited a + 3.65% and + 5.11% change in weight at 8 and 24 weeks, respectively. Therefore, the *APOA2* G allele was shown to be associated with an increasing trend in weight (*p* = 0.01 and *p* = 0.004 at 8 and 24 weeks, respectively; Fig. 3B). In regards to changes in fat mass, the *APOA2* AA group exhibited a − 7.47% (*p* < 0.001) and − 14.13% (*p* < 0.001) change in fat mass after 8 and 24 weeks, respectively, whereas the *APOA2* AG and *APOA2* GG groups presented a − 0.50% (*p* = 0.807) and − 4.31% (*p* = 0.084) and − 4.42% and + 12.05% change in fat mass, respectively, at 8 and 24 weeks. Therefore, the *APOA2* G allele was associated with a significant increase in fat mass at 24 weeks (*p* = 0.0035) but not after 8 weeks (Fig. 3C). Regarding dietary behaviors, the *APOA2* AA group exhibited a + 18.60% (*p* < 0.001) change in the proportion of healthy diet at 8 weeks, while the *APOA2* AG group showed a − 10.02% (*p* = 0.365) change. The *APOA2* GG group showed no change between the measurements taken at baseline and 8 weeks. Thus, the *APOA2* AA genotype significantly promoted healthy diet proportions after dCBT-O when compared to the AG and GG genotype at 8 weeks post-intervention (*p* = 0.036; Fig. 3D). Any of the results did not pass the strict multiple comparison corrected P threshold.

### *SNP* × *SNP interaction*

**Figure 4:**
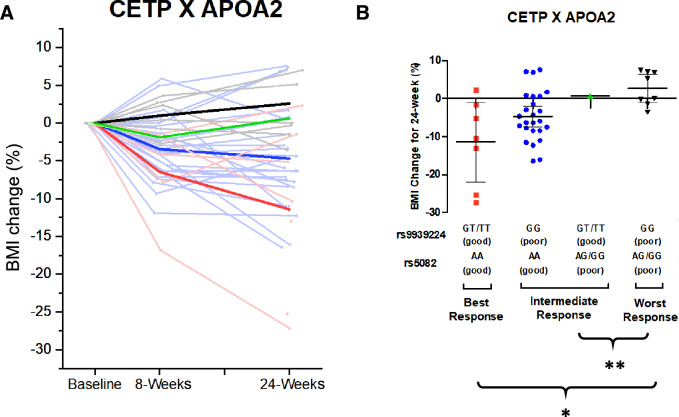
The effects of the interactions between *CETP* and *APOA2* on BMI change after dCBT-O; *p < 0.05; **p < 0.01.

To test for a gene–gene effect between the *CETP* and *APOA2* genotypes, they were classified into three interaction groups: best (good × good), intermediate (good × bad), and worst (bad × bad) response (shown in Fig. 4A,B). Linear regression analysis revealed that the primary outcome (BMI change) was significantly different between the three interaction genotype groups (*p* < 0.05). Moreover, the BMI change was significantly unfavorable in the worst-response group (+ 2.62%) when compared to both the intermediate (− 4.49%; *p* = 0.007) and best (− 11.45%; *p* = 0.038) response groups.

### Associations with other SNPs and phenotypes

Several other SNPs were also associated with changes in clinical outcomes (Supplementary Table S1). For example, BMI change at 24 weeks, which was the primary outcome, was significantly associated with *FTO* rs1421084 (*p* = 0.044), *LIPC* rs1800588 (*p* = 0.004), and *COMT* rs737865 (*p* = 0.049). Furthermore, several SNPs were associated with the baseline phenotypes (Supplementary Table S2). For instance, *MC4R* rs17782313 was associated with anthropometric (body weight, *p* = 0.003; BMI, *p* = 0.001; body fat percentage, *p* = 0.014) and psychological measures (depression; *p* = 0.048) at baseline.

## Discussion

This study is unique in that it integrates multi-dimensional components, including psychological elements, eating behavior phenotypes, and anthropometric measures, to determine the response to lifestyle modification (dCBT-O). Our findings suggest that the *CETP* rs9939224 SNP could predict “super-responders” which will exhibit greater BMI reduction (over -5% of change) after lifestyle modification, while *APOA2* rs5082 could predict “poor-responders” which will exhibit no BMI gain (over + 0.02% of change) after lifestyle modification (dCBT-O). Moreover, these SNPs may play a role in modulating changes in healthy eating behavior and psychological behavior during the intervention period. We also found that classification using gene–gene interaction between *CETP* rs9939224 and *APOA2* rs5082 predicts the best response associated with a greater decrease in BMI after lifestyle modification (dCBT-O).

*CETP*, which is a hydrophobic glycoprotein, plays a key role in transporting cholesterol from the peripheral tissues to the liver and is highly expressed in adipose tissue with low lipid contents^19,20^. According to previous studies, the *CETP* SNP rs3764261 was primarily associated with plasma high-density lipoprotein cholesterol (HDL-C), triglycerides (TG), and the risk of coronary atherosclerosis^20,21^. On the other hand, genetic variants of the *CETP* gene are also associated with alcohol consumption^22,23^ and dietary fat intake^24^. In addition, some findings have revealed the interactions between *CETP* polymorphism and dietary carbohydrate intake in relation to metabolic factors such as obesity and diabetes mellitus^25,26^. Furthermore, it has been reported that the *CETP* SNP rs3764261 is highly associated with HDL-C change after lifestyle modification intervention (i.e., Look AHEAD, Weight Gain Prevention; SNAP)^27,28^. The rs3764261 SNP is linked with the rs9939224 SNP evaluated in the present study with a linkage disequilibrium of D′ = 0.863^29^. In addition, our study revealed that the *CEPT* SNP rs9939224 modulates changes in BMI, healthy eating behavior, and psychological behavior after lifestyle modification (dCBT-O). Accordingly, the group considered super-responders based on this *CETP* SNP can be preferentially recommended to receive lifestyle modification treatments (dCBT-O). These results emphasize how this specific SNP significantly influences the behavioral and psychological mechanisms associated with the efficacy of obesity treatments.

*APOA2* is a protein involved in TG, fatty acid, and glucose metabolism^30^. Previous studies have reported that it is closely associated with insulin resistance, obesity, and hypertriglyceridemia. Moreover, the G genetic variants within the *APOA2* promoter (rs5082) are associated with higher food consumption and lower polyunsaturated fatty acid (PUFA) intake^31,32^. The G allele in the *APOA2* promoter, which is generally associated with lower *APOA2* expression, would give rise to lower plasma concentrations of *APOA2*^33,34^. Thus, since *APOA2* also acts as a satiety signal, lower plasma *APOA2* concentrations lead to a greater appetite^31,32^. The effect of the G allele on appetite could be the underlying biological mechanism linking this genotype to poor treatment response after lifestyle modification. Notably, this is the first study to show that the *APOA2* rs5082 SNP can be used as a precision medicine biomarker to predict the efficacy of an intervention. Our novel findings have shown that the *APOA2* rs5082 SNP modulates BMI and healthy eating behavior changes after lifestyle modification (dCBT-O). These results suggest that those who exhibit this SNP may have a higher chance of becoming poor responders to lifestyle modification (dCBT-O) can consider receiving other treatments, such as pharmacotherapy or surgical therapy.

Gene–gene interactions are essential to maximize the clinical efficacy of precision medicine, especially when single gene predictions have limited efficacy^35^. Thus, we also investigated combinations between *CETP* and *APOA2* SNPs to determine potential associations which may influence the response to interventions. The interactions between *CEPT* and *APOA2* SNPs showed augmented predictive power among the groups (− 11.45% mean BMI change for the best response group vs. + 2.62% mean BMI change for the worst response group), which were associated with changes in obesity-related phenotypes after lifestyle modification (dCBT-O). Therefore, these results suggest that gene–gene interactions between *CETP* and *APOA2* SNPs could be significant determinants of the clinical efficacy of lifestyle modifications (dCBT-O).

The strengths of this study include the use of randomized controlled trial data from lifestyle modification interventions (dCBT-O). We also analyzed multidimensional phenotypes, which allowed for a more comprehensive analysis of predictors of treatment responses. Furthermore, conducting an analysis of gene–gene interactions between *CETP* and *APOA2* clearly showed the augmented prediction efficacy of these SNPs in regards to the outcomes of lifestyle modification interventions (dCBT-O). This study also has several limitations. Firstly, we limited the sample size by restricting our study to the lifestyle modification (dCBT-O) arm. As a result, there was only one subject with the GG genotype of the *APOA2* SNP based on our analysis. With that said, our findings must be interpreted with caution. In addition, only Korean participants made up the total number of participants; therefore, more research is required to determine whether our findings apply to other ethnic groups. Secondly, the risk of false positives (type I error) should be considered due to the multiple comparison issues. Regarding this issue, we applied Bonferroni correction for multiple comparisons. After this correction, there were no results passing the strict multiple comparison corrected P threshold. However, given that there is tight linkage disequilibrium among the analyzed SNPs and strong associations between the phenotypes, the Bonferroni correction method may be overly conservative. This may result in a higher chance of false negative (type II error). Overall, these results indicate that a larger sample size is required to adjust the findings from this study. Thirdly, since the study population was limited to women with a BMI > 24 kg/m^2^, aged between 19 and 39 years, and with relatively high motivation levels, this limits the extrapolation of the results of our study to the general population. Lastly, in terms of our study protocol, the total number of visits by participants was only three times during the 24 weeks of the study, which may be insufficient. Furthermore, we did not include some important weight loss factors, such as physical activity levels, as our measurements. Thus, we propose that future studies assess the measures more frequently and include the determining factors of weight management.

## Methods

### Study design and participants

This study was an open-label, randomized controlled trial approved by the Institutional Review Board of Seoul National University Hospital (approval number H-1707-122-872). The procedures were followed in accordance with the Helsinki Declaration of 1975 as revised in 1983. The aim of this study was to investigate genetic predictors of the dCBT-O. Thus, we determined that dCBT-O led to successful lifestyle modifications, thereby enabling participants to manage a healthy weight. The data which was examined in the current analysis focused on participants in the dCBT-O group which included 45 participants in total. To justify the sample size for this study, we assumed that individuals with dominant and recessive genes per genotype have a 3:1 ratio when comparing the mean between genotype groups. Setting the effect size (d) at 1, and a desired statistical power of 0.8, our calculated total sample size was estimated to be 44. The detailed design and methods of the trial are published elsewhere^18^. Briefly, the participants in the dCBT-O group were given daily feedback and assignments from a psychologist based on the CBT modules for 8 weeks and were able to access the digital tools from the start of the intervention period until the 24-week follow-up.

### Assessments

The primary outcome of this study was a change in body mass index (BMI) and other measures, such as body weight and body fat mass, were also assessed using the InBody H20B analyzer. Healthy eating behavior measures (healthy diet intake, healthy diet proportion, and healthy diet diversity) were measured using a buffet test-meal^36^. In addition, the Dutch Eating Behavior Questionnaire (DEBQ) was used to determine psychologically based eating behaviors, namely restrained eating (DEBQ-RE), emotional eating (DEBQ-EM), and external eating (DEBQ-EX). We also tested blood samples after a 10-h fast. Detailed descriptions of the measurements have been published elsewhere^18^.

### Intervention

The dCBT-O was a multi-dimensional, daily based, individualized coaching system delivered by a psychologist applying CBT modules within a digital platform. The therapist monitored and assessed multiple elements representing the behavior, cognition, mood, and motivation of each individual in the dCBT-O group. This intervention aimed to promote healthy eating behaviors through lifestyle modification. Further explanations of the intervention are presented elsewhere^18^.

### Genetic analysis

Blood DNA was extracted using the ExgeneTM Tissue SV (GeneAll, Seoul, Korea). All DNA samples were amplified and randomly portioned into 25–125 bp fragments, which were in turn purified, re-suspended, and hybridized with the Theragen Precision Medicine Research Array (Theragen PMRA array), which is a customized array based on the Asian Precision Medicine Research Array (Thermo Fisher Scientific, Waltham, Massachusetts, USA). Following hybridization, the bound target was washed under stringent conditions to remove non-specific background signals and minimize noise resulting from random ligation events. Subsequently, we genotyped 820,000 SNPs using the Theragen PMRA array, according to the manufacturer’s instructions, therefor obtaining genome-wide coverage in five major populations, as well as imputation accuracy for GWAS markers of 0.90 and 0.94 with minor allele frequencies (MAF) of > 1% and > 5% for 7.4 million imputed markers in an Asian population. To reduce potential concerns regarding batch effects and the possibility of false associations, we applied highly stringent quality control measures to select SNPs for use in the case and control datasets. Quality control procedures were performed on each of the 820,000 SNPs before the association tests were conducted. The SNP set was filtered based on the genotype call rates (≥ 0.95) and MAF (≥ 0.10). The Hardy–Weinberg equilibrium (HWE) was calculated for individual SNPs using an exact test. All the SNPs reported in this manuscript were shown to have HWE *p*-values of > 0.01. After filtering, 560,795 polymorphic SNPs were analyzed on chromosomes 1 through 22. The 97 obesity-related SNPs of the GENESTYLE MEDIFIT were selected based on previous reports that found a significant association more than twice, while the gene function and mechanisms were well established by previous molecular studies (see Supplementary File S3).

### Statistical analysis

Statistical analysis was performed using the SPSS software (IBM, Armonk, New York, United States) version 23.0. The outcomes were adjusted by their corresponding baseline values by using analysis of change (percent change from baseline), to encounter the problem related to the regression of the mean^37^. A *p*-value of < 0.05 was considered statistically significant for all two-sided tests and multivariate comparisons. For multiple comparison corrections, threshold of p < 0.0001 was used (P threshold of 0.05 divided by 380, corresponding to eighty-three SNPs and two-hundred ninety-seven phenotypes). Linear regression analysis was used to examine the additive effects of each SNP on anthropometric, eating behavioral, psychological, and physiological measures. To verify the interaction between SNPs in the identified model, an ANOVA test was performed. We applied Welch’s test and the Games-Howell test for post hoc analysis to address violations in Levene’s test. In addition, we analyzed the data while considering imputation and excluded those samples with only baseline data.

### Ethics approval and consent to participate

All participants provided written, informed consent for participation in the study. The Institutional Review Boards of Seoul National University Hospital approved the consent documents, and the study was conducted according to the principles of ethical research practice outlined by the Declaration of Helsinki.

## Conclusion

The results of our study revealed for the first time that genotypes (*CETP*; rs9939224 and *APOA2*; rs5082) can predict the clinical outcomes and multi-dimensional components, such as eating behaviors (healthy diet proportion and diversity change) and psychological characteristics (emotional and restrained eating behaviors), of lifestyle modification interventions (dCBT-O). In addition, we identified a practical gene–gene interaction that could predict super-responders to the intervention. Thus, our results demonstrate that *CETP* and *APOA2* SNPs can be suggested as key elements for genotype-based precision medicine for obesity.

### Supplementary Information

Supplementary Table 1.

Supplementary Table 2.

Supplementary Table 3.

## Data Availability

The data that support the findings of this study are freely available at the following link: https://github.com/meelim/dcbtsnp.

## Abbreviations

SNP: Single nucleotide polymorphism
dCBT-O: Digital cognitive behavior therapy for obesity
CETP: Cholesteryl ester transfer protein
APOA2: Apolipoprotein A-II
BMI: Body mass index
DEBQ: Dutch eating behavior questionnaire
DEBQ-RE: Dutch eating behavior questionnaire: restrained eating
DEBQ-EX: Dutch eating behavior questionnaire: external eating
DEBQ-EM: Dutch eating behavior questionnaire: emotional eating
HWE: Hardy–Weinberg equilibrium
